# hTERT phosphorylation by PKC is essential for telomerase holoprotein integrity and enzyme activity in head neck cancer cells

**DOI:** 10.1038/sj.bjc.6603008

**Published:** 2006-02-28

**Authors:** J T Chang, Y-C Lu, Y-J Chen, C-P Tseng, Y-L Chen, C-W Fang, A-J Cheng

**Affiliations:** 1 Department of Radiation Oncology, Chang Gung Memorial Hospital, Taoyuan 333, Taiwan; 2 Graduate Institute of Medical Biotechnology, Chang Gung University, 259 Wen-Hwa 1st Road, Taoyuan 333, Taiwan

**Keywords:** hTERT, PKC isoenzymes, phosphorylation, telomerase

## Abstract

Telomerase activity is suppressed in normal somatic tissues but is activated in most cancer cells. We have previously found that all six telomerase subunit proteins, including hTERT and hsp90 are needed for full enzyme activity. Telomerase activity has been reported to be upregulated by protein kinase C (PKC), but the mechanism is not clear. In this study, we examined how PKC regulates telomerase activity in head and neck cancer cells. PKC inhibitor, bisindolylmaleimide I (BIS), inhibited telomerase activity but had no effect on the expressions of telomerase core subunits. RNA interference (RNAi) and *in vitro* phosphorylation studies revealed that PKC isoforms *α*, *β*, *δ*, *ε*, *ζ* specifically involved in telomerase regulation, and the phosphorylation target was on hTERT. Treatment with the hsp-90 inhibitor novobiocin dissociated hsp90 and hTERT as revealed by immunoprecipitation and immunoblot analysis and reduced telomerase activity. Treatment with the PKC activator SC-10 restored the association of hsp90 and hTERT and reactivate telomerase, suggesting that hTERT phosphorylation by PKC is essential for telomerase holoenzyme integrity and function. Analysis on clinical normal and tumour tissues reveal that the expressions of PKC *α*, *β*, *δ*, *ε*, *ζ* were higher in the tumour tissues, correlated with telomerase activity. Disruption of PKC phosphorylation by BIS significantly increased chemosensitivity to cisplatin. In conclusion, PKC isoenzymes *α*, *β*, *δ*, *ε*, *ζ* regulate telomerase activity in head and neck cancer cells by phosphorylating hTERT. This phosphorylation is essential for telomerase holoenzyme assembly, leading to telomerase activation and oncogenesis. Manipulation of telomerase activity by PKC inhibitors is worth exploring as an adjuvant therapeutic approach.

Telomerase is a specialised ribonucleoprotein enzyme responsible for synthesising telomeric DNA at the end of chromosomes. Human telomeres consist of hundreds to thousands of tandem repeats of the sequence TTAGGG, which is essential for stabilising the chromosome (Morin, 1989). The telomere length is progressively shortened during cell replication, which is thought to be an indicator of cell senescence. Telomerase activity is undetectable in normal somatic cells, whereas telomere length is stabilised and telomerase activity is detected in about 85% of cancer cells. Reactivation of telomerase is thus believed to be involved in cellular immortalisation and tumorigenesis (Counter *et al*, 1992; Kim *et al*, 1994).

Telomerase is a holoenzyme consisting of several subunits including hTR (human telomerase RNA), TEP1 (telomerase-associated protein1), hTERT (human telomerase reverse transcriptase), hsp90 (heat shock protein 90), p23, and dyskerin. hTR functions as a template for telomere elongation (Feng *et al*, 1995). TEP1 is thought to be associated with RNA and protein binding (Nakayama *et al*, 1997). hTERT contains reverse transcriptase motifs and functions as the catalytic subunit of telomerase (Linger *et al*, 1997), while hsp90 and p23 are molecular chaperons which bind to hTERT and contribute to telomerase activity (Holt *et al*, 1999). Dyskerin is thought to mediate the interaction with telomerase ribonuclear protein and facilitate hTR processing or assembly to form an active telomerase complex (Mitchell *et al*, 1999). Our previous study has shown that hTERT is regulated, whereas the other components are expressed more constantly. Although hTERT has a rate-limiting effect on enzyme activity, the other telomerase subunits all participate in full enzyme activity (Chang *et al*, 2002).

Several mechanisms by which telomerase activity is regulated have been reported. At the transcriptional level, transcriptional activators (c-*myc*, Sp1) and repressors (Mad1) regulate hTERT expression and telomerase activity (Wu *et al*, 1999; Gunes *et al*, 2000; Kyo *et al*, 2000). Akt, c-Abl, and protein kinase C (PKC) have all been shown to contribute to post-transcriptional regulation of the enzyme activity by kinase phosphorylation (Kang *et al*, 1999; Kharbanda *et al*, 2000; Yu *et al*, 2001). Our previous study found that PKC regulated telomerase activity in nasopharyngeal cancer cells (Ku *et al*, 1997). Recent studies on telomerase regulation by PKC have shown that telomerase activation can be achieved by both transcriptional and post-transcriptional effects on hTERT. It has been demonstrated that telomerase in human breast cancer cells is regulated by PKC *α* through phosphorylation of hTERT (Li *et al*, 1998). PKC *ζ* has been reported to regulate telomerase activity through both transcription and post-transcriptional mechanisms in nasopharyngeal cancer cells and peripheral T lymphocytes during T-cell activation (Yu *et al*, 2001; Sheng *et al*, 2003). These results indicate that telomerase is regulated by various PKC isoenzymes, perhaps depending on the specific cell type or cell status.

Although several reports demonstrate the role of PKC in telomerase regulation, exactly how it functions in different types of cells and in carcinogenesis remains largely unknown. In this study, we examined whether PKC regulates telomerase activity in head and neck cancer cells and which PKC isoforms may be involved. Since the integrity of telomerase holoenzyme is important for telomerase full activity, we investigated whether PKC plays a part in telomerase holoprotein structure.

## MATERIALS AND METHODS

### Chemicals

PKC inhibitor bisindolylmaleimide I (BIS), PKC activator SC-10 and hsp90 inhibitors novobiocin were all purchased from Calbiochem (San Diego, CA, USA). BIS has been reported to inhibit PKC function by competition for ATP binding, leading to the failure of PKC phosphorylation on its substate (Yu *et al*, 2001). SC-10 is a potent Ca^2+^-dependent PKC activator (Watson *et al*, 1992).

### Patients and tissue samples

Human tissues used for this study were obtained from patients with head and neck squamous cell carcinoma, admitted to the Otorhinolaryngology or Head and Neck Surgery clinics at Chang Gung Memorial Hospital (Taoyuan, Taiwan). Written informed consent was obtained from all participating patients. Biopsies of cancer and grossly normal mucosal tissues were obtained from each subject before chemo- or radiotherapy. A portion of each tissue sample was stored in liquid nitrogen until use for molecular assay.

### Cell culture, chemical treatment, and determination of cell viability

The OEC-M1 oral cancer cell line was used (Yang *et al*, 2001). The cells were cultured routinely in RPMI-1640 medium (Gibco BRL, Rockville, MD, USA) supplemented with 10% fetal calf serum and 1% antibiotics at 37°C in a humidified incubator containing 5% CO_2_. OEC-M1 cell were seeded at a density of 1.2 × 10^6^ per 100-mm dish in complete medium. When the cells reached 60% confluence, they were treated with the various experimental chemicals. Cell viability was determined by staining with 0.25% trypan blue, with the fraction of stain-negative cells taken as the surviving fraction.

### Cellular protein extraction and analysis of telomerase activity

Cell pellets were suspended in lysis buffer (10 mM Tris-HCl, pH 7.4, 1 mM MgCl_2_, 1 mM EGTA, 0.5% CHAPS, 10% glycerol, 0.1 mM PMSF, 10 *μ*l ml^−1^ aprotinin, 10 *μ*g ml^−1^ leupeptin) and incubated for 30 min at 4°C while being gently mixed. After centrifuging at 14 000 rpm for 30 min at 4°C, the supernatant was transferred to fresh tubes for the telomerase activity assay. Protein concentrations were determined using Coomassie Protein Assay Reagent (Bio-Rad, CA, USA).

Telomerase activity was assayed with the telomeric repeat amplification protocol-enzyme immunoassay (TRAP-EIA) as we have previously described (Cheng *et al*, 1999). Telomerase activity was determined based on the ability to produce telomere repeats by using a PCR-based TRAP assay and measuring the PCR products using an EIA-based assay. Briefly, 0.3 *μ*g of protein extract was added to a TRAP reaction buffer and incubated at 25°C for 15 min, followed by amplification by 30 cycles of PCR at 94°C for 30 s, 55°C for 30 s, and 72°C for 1 min. After PCR, 5 *μ*l of the PCR products were placed in streptavidin-coated wells and incubated with 200 *μ*l of antidigoxigenin antibody conjugated with horseradish peroxidase (10 mU ml^−1^) at 37°C for 30 min in EIA reaction buffer. After washing, enzyme reactions were initiated by the addition of 200 *μ*l of tetramethylbenzidine substrate solution to each well. After 10 min, the reactions were stopped by the addition of 50 *μ*l of 2 N HCl to each well. Colorimetric signals were determined by measuring the absorbance at 450 nm using an automatic microwell reader.

### RNA extraction and assay for telomerase subunit expressions

The gene expression of each telomerase subunit (TEP1, hTERT, hsp90 and p23) was analysed using a reverse transcriptase–polymerase chain reaction (RT–PCR) assay that we have previously described (Chang *et al*, 2002). Total RNA from cells was isolated with TRIzol reagent (Gibco BRL) following the manufacturer's instructions. The concentration, purity, and amount of total RNA were determined by ultraviolet spectrophotometry. The reverse transcription reaction was performed by incubation of a reaction mixture containing 300 ng RNA, 100 pmole of poly-T oligonucleotide, 4 U of reverse transcriptase, 10 units of RNase inhibitor, and 25 mM dNTP in a total of 30 *μ*l reaction buffer at 37°C for 1 h. PCR reactions were carried out with 30 cycles of denaturation at 94°C for 40 s, annealing at 56°C for 40 s and extension at 72°C for 1 min. The PCR products were analysed by 2% agarose gel electrophoresis, stained with ethidium bromide, and visualised and photographed by illuminating with 254 nm UV. The photograph was scanned and the band intensities were measured using densitometry.

### Cytosol and nuclear protein extraction

Cell pellets were resuspended in 400 *μ*l of cold buffer (20 mM Hepes, pH 7.9, 150 mM NaCl, 10% glycerol, 1 mM EDTA, 1 mM EGTA, 1 mM DTT, and 0.5 mM PMSF) on ice for 15 min. After adding 25 *μ*l of 10% Nonidet P-40, cells were vortexes for 10 s and centrifuged at 14000 rpm for 1 min. The supernatant was transferred to a fresh tube as the cytosolic fraction. To fractionate nuclear protein, the nuclear pellet was resuspended in 50 *μ*l ice-cold buffer (20 mM Hepes, pH 7.9, 0.4 M NaCl, 1 mM EDTA, 1 mM EGTA, 1 mM DTT, and 1 mM PMSF). After incubation at 4°C for 15 min with vigorously shaking, the nuclear extract was centrifuged at 14 000 rpm for 5 min. The supernatant thus contained nuclear protein, the concentration of which was determined with Coomassie Protein Assay Reagent (Bio-Rad, CA, USA).

### Immuoprecipitation and immunoblot analysis

The immunoprecipitation and immunoblot methods used were similar to the protocols suggested by the manufacturer (Santa Cruz Biotech, CA, USA). Briefly, prior to immunoprecipitation, 30 *μ*l protein A/protein G sepharose beads (Santa Cruz Biotech) conjugated with 4 *μ*g of specific antibodies (goat polyclonal anti-hTERT, clone SC-7214 and mouse monoclonal anti-hsp90, Santa Cruz Biotech) were washed with 1 ml RIPA buffer (20 mM Tris-HCl, pH 7.4, 150 mM NaCl, 0.5% Nonidet P-40, 0.1% SDS, 1% deoxycholate) followed by 1 ml blocking solution (5% BSA, 0.02% calf thymus DNA, 0.3% CHAPS). For immunoprecipitation, 1 mg of the cellular or nuclear protein extract was incubated with 30 *μ*l conjugated protein A/protein G sepharose beads and incubated while rotating for 4 h at 4°C. The beads were collected by centrifugation at 3000 rpm for 5 min at 4°C and washed three times with 0.6 ml of cool RIPA buffer. Finally, the beads were collected by centrifugation at 3000 rpm for 5 min at 4°C, resuspended in 20 *μ*l of a sample buffer (25 mM Tris-HCl, pH 6.8, 10% glycerol, 2% SDS, 5% *β*-mercaptoethanol), and subjected to immunoblot analysis. Beads conjugated with nonimmunised goat serum IgG were used as a negative control to determine the specific effect of immunoprecipitation.

For immunoblot analysis, cellular protein extract was used for determination of PKC isoenzymes, and nuclear protein extract was used for examination of hTERT level. Total of 20 *μ*g of proteins in the sample buffer or 3 *μ*l of the immunoprecipitated samples were prepared. All samples were boiled at 95°C for 5 min, and subjected to 10% SDS-polyacrylamide gel for electrophoresis. The protein image on the electrophoretic gel was transferred to a nitrocellulose membrane and blocked with 5% nonfat milk in PBST solution (phosphate buffer saline plus 0.1% Triton X-100). After being washed twice with PBST, the membrane was incubated with a proper dilution of first antibodies (for PKC isoforms: PKC *α*, *β*I, *β*II, *γ*, *δ*, *ε*, *ζ* and *η*, Santa Cruz Biotechnology; and for pan-PKC phosphosubstrate, Cell Signaling, New England BioLabs, Beverly, MA, USA) at room temperature for 2 h. The membrane was washed again and incubated with anti-mouse IgG or anti-rabbit IgG antibody conjugated with horseradish peroxidase. The membrane was treated with ECL developing solution (Amersham Pharmacia Biotech, New Territories, Hong Kong) and exposed to X-ray film. Actin expression was used as an internal control to determine the relative expression of PKC isoenzymes.

### *In vitro* phosphorylation assay

A total of 20 *μ*g of BIS-treated cell or nuclear lysates was mixed with 25 ng of a specific PKC isoenzyme (Upstate biotechnology, NK, USA) in a final volume of 25 *μ*l containing 6 *μ*l of assay dilution buffer (40 mM MOPS, pH 7.2, 50 mM *β*-glycerophosphate, 2 mM sodium orthovandate, 2 mM dithiothreitol, 2 mM CaCl_2_), 5 *μ*l lipid activator (0.5 mg ml^−1^ phosphotidylserine, 0.05 mg ml^−1^ diacylglycerol) and 5 *μ*l ATP mixture (75 mM MgCl_2_, 500 *μ*M ATP, 100 *μ*Ci [*γ*-P^32^]-ATP). After incubation at 30°C for 10 min, cellular proteins were extracted for analysis. To determine the effect of PKC phosphorylation on telomerase activity, TRAP-EIA assay was performed as described above. To determine whether hTERT is the PKC phosphorylation target, anti-hTERT antibodies were used to immunoprecipitate the phosphorylated protein. After SDS–PAGE separating the precipitated proteins, the gel was subjected to autoradiography.

### RNA interference cloning and cellular transfection

pTOPO-U6 vector was used for PKC-RNAi construction as we have described previously (Tseng *et al*, 2003). RNAi oligonucleotides for PKC *α*, *β*, *γ*, *δ*, *ε*, *ζ*, and *η* RNAi are listed in Table 1. The RNAi oligonucleotides were annealed and ligated to pTOPO-U6 vector corresponding to the blunt end and the overhang that matched the *Eco*RV- and *Bbs*I-digested pTOPO-U6. The ligation between the annealed oligonucleotides and pTOPO-U6 at the *Eco*RV and *Bbs*I cloning sites generated PKC-RNAi plasmid. OEC-M1 cells were transfected with a mixture of 3 *μ*g plasmid DNA and 3 *μ*l Lipofectamin™ 2000 (Invitrogen, CA, USA) in 3 ml OPTI-MEM medium (Gibco) and incubated at 37°C in 5% CO_2_ for 12 h. After transfer to complete culture medium, the cells were continuously incubated for 1–3 days. Cellular proteins were determined by immunoblot and telomerase activity was measured by TRAP-EIA assay.

## RESULTS

### BIS inhibited telomerase activity but had no effect on gene expression

**Figure 1:**
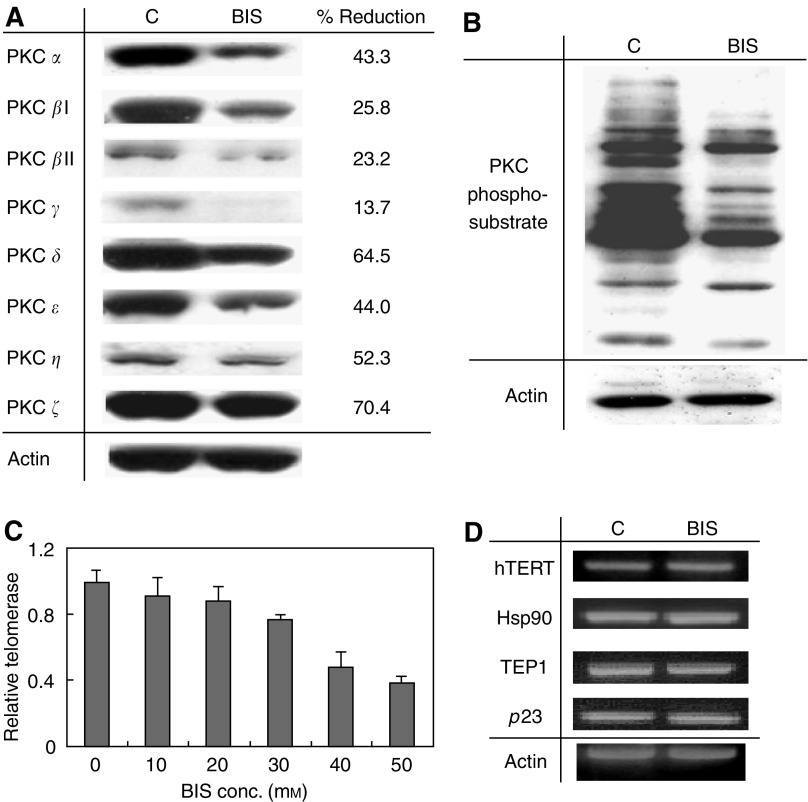
Effects of PKC inhibitor, BIS, on telomerase activity and the telomerase subunit expressions. OEC-M1 cells were treated with 40 *μ*M (**A**, **B**, **D**) or various amounts (**B**) of BIS for 48 h. (**A**) Effect of BIS on PKC isoenzyme expressions. Eight PKC isoenzymes (*α*, *β*I, *β*II, *γ*, *δ*, *ε*, *ζ*, *η*) were determined by immunoblot, as indicated at the left of the figure. (**B**) Effect of BIS on PKC phosphorylation activity. PKC activity was determined by examining the amount of PKC-phosphosubstrate using pan-antibody by immunoblot analysis. C: Control, without drug treatment; BIS: BIS-treated cells. (**C**) Effect of BIS on telomerase activity. Telomerase activity was determined by TRAP-EIA as described in the materials and methods. (**D**) Effect of BIS on the expressions of telomerase subunits. Telomerase subunits, hTERT, hsp90, TEP1 and p23 were determined by RT–PCR as indicated on the left of the figure. Actin expression was determined as an internal control.

To investigate the mechanism by which PKC regulates telomerase activity leading to cancer formation, the PKC specific inhibitor BIS was used. In this study, we first examined the effects of BIS in OEC-M1 cells. OEC-M1 cells were treated with various amount of BIS (0 to 50 *μ*M) for 48 h. Cellular proteins were extracted for the determination of PKC protein expressions and activity. The expression of PKC isoenzymes *α*, *β*I, *β*II, *γ*, *δ*, *ε*, *ζ*, and *η* was determined by immunoblot analysis using specific PKC isoenzyme antibodies. Figure 1 shows the representative results of cells treated with 40 *μ*M of BIS. At this dose, cell viability is approximately 75%. As shown in Figure 1A, all PKC isoenzymes were reduced in BIS-treated cells, ranging from 13% (PKC-*γ*) to 70% (PKC-*ζ*) reduction. Enzymatic activity was determined by examining the amount of PKC- phosphosubstrates by immunoblot analysis using anti-PKC phosphosubstrate antibody. As shown in Figure 1B, less phosphosubstrate was detected in BIS-treated protein extract, indicating decreased PKC activity in BIS-treated cells. These results indicated that BIS inhibits PKC protein expression as well as enzyme activity.

The effect of BIS on telomerase activity and gene expression were examined. OEC-M1 cells were incubated with various amount of BIS (0 to 50 *μ*M) for 48 h and the telomerase activity was determined by TRAP-EIA assay. As shown in Figure 1C, telomerase activity was inhibited in a dose-dependent manner by BIS, with an approximately 50% decrease in telomerase activity induced by treatment with 40 *μ*M of BIS. In order to examine whether the loss of telomerase activity resulted from the suppression of telomerase gene expression, transcriptional levels of the telomerase core subunit genes hTERT, TEP1, hsp90 and p23 were determined by RT–PCR. As shown in Figure 1D, BIS had no effect on core telomerase gene expression. This indicates that PKC-inhibited telomerase activity in head and neck cancer cells does not occur through the suppression of gene expression. It more likely based on a post-transcriptional regulatory mechanism.

### PKC *α*, *β*, *δ*, *ε*, and *ζ* are involved in telomerase regulation through phosphorylation mechanism

**Figure 2:**
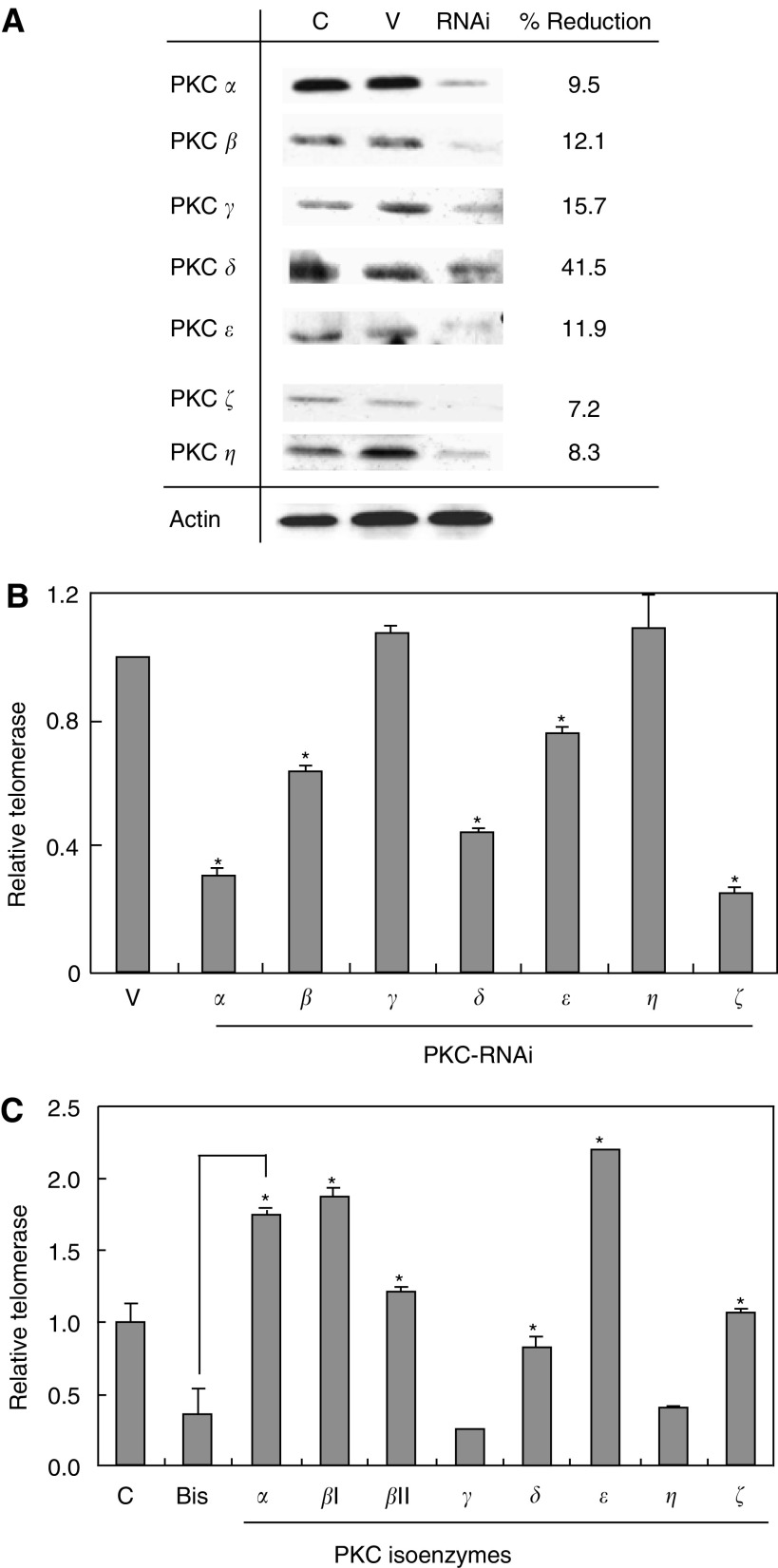
Effects on telomerase activity by specific PKC isoenzymes. (**A**) OEC-M1 cells were transfected with specific PKC-RNAi plasmids for 48 h. Immunoblot analysis to determine the protein expression levels of each PKC isoenzyme. Actin gene expression was measured as an internal control. Actin expression remained consistent in every transfected sample. In this figure, the results of PKC *α*-RNAi transfection are shown as representative of all the RNAi experiments. C: control cells without plasmid transfection, V: cells transfected with vector, RNAi: cells transfected with specific PKC-RNAi plasmid as indicated on the left of the figure. (**B**) Telomerase activity was determined after transfection with specific PKC-RNAi plasmids as indicated on the bottom of the figure. (**C**) OEC-M1 cells were treated with 40 *μ*M BIS for 48 h. Cell lysates were subjected to *in vitro* phosphorylation by specific PKC isoenzymes as indicated, followed by determination of telomerase activity. ^*^Statistical significance using Student's *t*-test (*P*<0.05 of two-sided test).

To examine which PKC isoenzyme is involved in telomerase regulation, telomerase activity was determined after specific suppression of PKC protein expression by RNA interference (RNAi). OEC-M1 cells were transfected with specific PKC-RNAi plasmid for 48 h and the cellular protein levels were determined by immunoblot. As shown in Figure 2A, all seven PKC isoenzymes (*α*, *β*, *γ*, *δ*, *ε*, *ζ* and *η*) were successfully suppressed by specific RNAi. Telomerase activity, however, was only inhibited by PKC *α*, *β*, *δ*, *ε* and *ζ* suggesting that these PKC isoenzymes involved in telomerase regulation (Figure 2B). To further confirm this observation as well as the mechanism of BIS suppression on telomerase activity, an *in vitro* phosphorylation experiment was performed. OEC-M1 cells were treated with 40 *μ*M BIS for 48 h. Cellular protein was extracted and subjected to *in vitro* phosphorylation by specific PKC isoenzymes, followed by determination of telomerase activity. As shown in Figure 2C, PKC-*α*, *β*I, *β*II, *δ*, *ε* and *ζ* but not *γ* and *η*, restored BIS-suppressed telomerase activity, consistent with the finding in the RNAi experiments. In fact, these isoenzymes produced a even higher level of enzyme activity than the control. This may be because endogenous telomerase in OEC-M1 cells was not fully activated. Phosphorylation by exogenous PKC may confer full enzyme activity, including the inactivated telomerase molecules present in the cells.

### hTERT was the target of PKC phosphorylation

**Figure 3:**
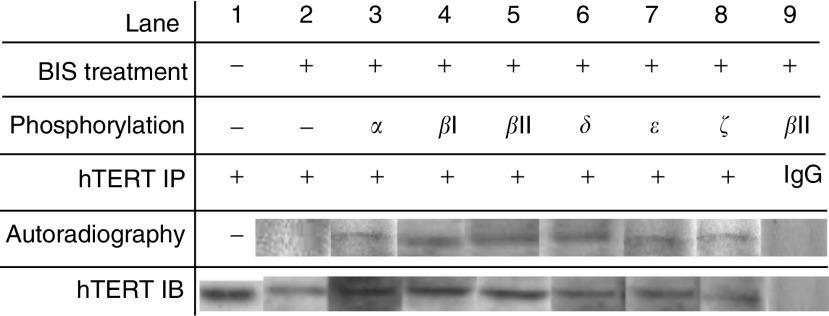
*In vitro* phosphorylation study for the target molecule hTERT. Nuclear proteins were phosphorylated using [*γ*-P^32^]-ATP by specific PKC isoenzymes as indicated on the top of the figure. Protein samples were immunoprecipitated by hTERT antibody. Samples were then subjected to autoradiography or immunoblot analysis. Proteins without phosphorylation (lanes 1 and 2) or immunoprecipitated with pre-immune goat IgG (IgG) (lane 9) were used as negative controls. An immunoblot of hTERT for each sample served as an internal control. Autoradiography data for immunoprecipitated samples demonstrate that hTERT molecules were phosphorylated by the PKC isoenzymes.

Since, we and others have previously demonstrated that hTERT is a regulated, rate-limiting telomerase subunit protein, we examined whether it was a target of PKC phosphorylation. Nuclear proteins from OEC-M1 cells were phosphorylated *in vitro* by specific PKC isoenzymes *α*, *β*I, *β*II, *δ*, *ε*, *ζ* using [*γ*-P^32^]-ATP. Protein samples were immunoprecipitated by hTERT antibody. Samples were then subjected to immunoblot analysis or autoradiography. Results are shown in Figure 3. In each sample, the protein immunoblotted by hTERT antibody served as an internal control to ensure the presence of hTERT protein in the samples. Proteins without phosphorylation (lanes 1 and 2) or those immunoprecipitated by preimmune goat IgG (lane 9), which had no detectable band on autoradiography, served as a negative control. The autoradiography data (lanes 3–8) indicate that all PKC phosphorylated proteins were immunoprecipitated by hTERT, indicating that hTERT is the target of PKC phosphorylation. Taken together, all the above studies indicate that PKC phosphorylates hTERT and is thus responsible for telomerase enzyme activity.

### PKC phosphorylation was essential for telomerase holoprotein integrity and telomerase activity

**Figure 4:**
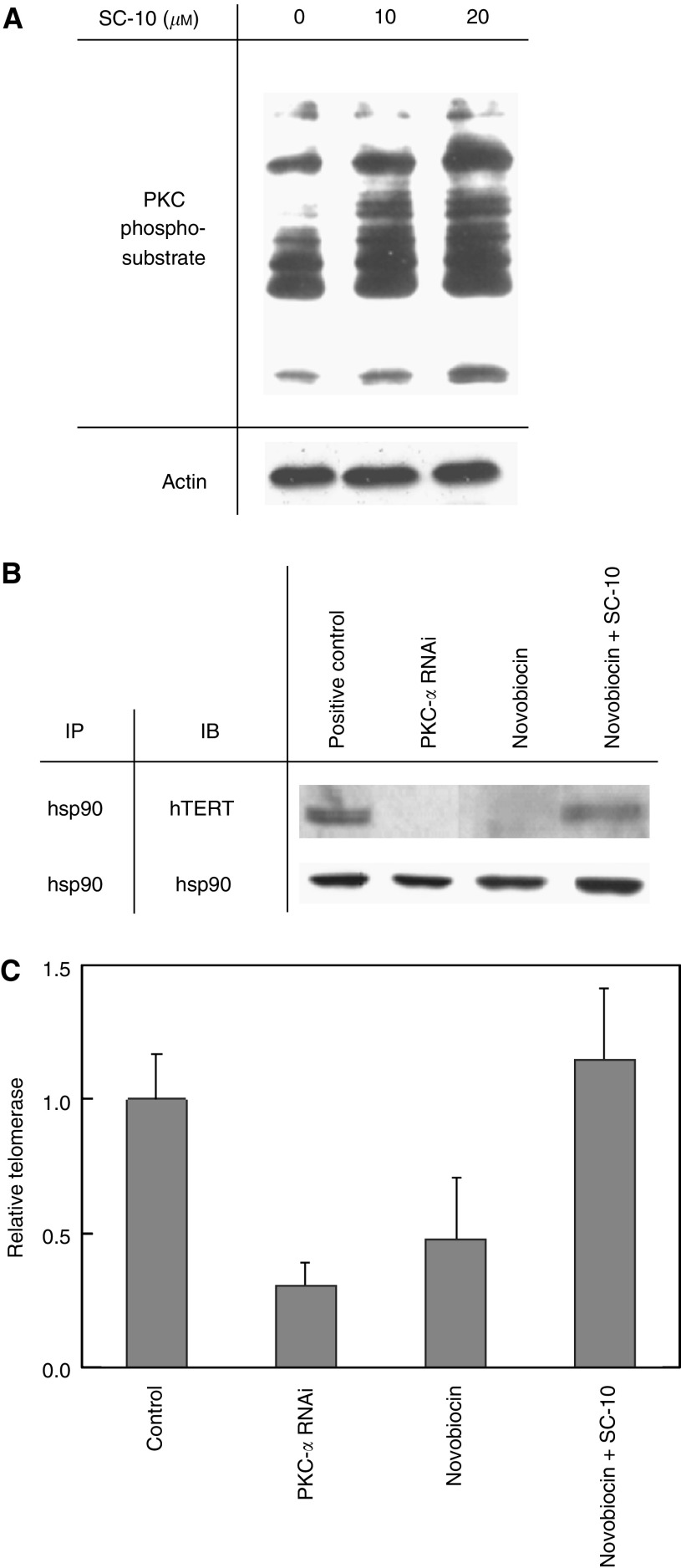
Effect of PKC phosphorylation on telomerase holoprotein integrity and the enzyme activity. (**A**) After treatment with 10 *μ*M SC-10, PKC activity was determined by examining the amount of PKC-phosphosubstrates using pan-antibody immunoblot analysis. (**B**) Association study of hTERT-hsp90 and the influence of PKC phosphorylation. Lanes 1 and 2: Nuclear proteins from OEC-M1 cells with or without PKC-RNAi plasmid transfection were extracted and subjected to immunoprecipitation and immunoblot. Lanes 3 to 4: OEC-M1 cells were treated with 300 *μ*M novobiocin for 24 h to disrupt the hsp-hTERT association. Cells were harvested or continuously cultured with 10 *μ*M SC-10 for an additional 24 h (novobiocin+SC-10). Nuclear proteins were subjected to immunoprecipitation by hsp90 followed by immunoblot by hTERT or hsp90 (as control). (**C**) Alterations of telomerase activity after hTERT-hsp90 disruption and reassociation. Lanes 1 and 2: Cellular proteins from OEC-M1 cells with or without PKC-RNAi plasmid transfection were extracted for determination of telomerase activity by TRAP-EIA. Lanes 3 to 4: OEC-M1 cells were treated with 300 *μ*M novobiocin for 24 h to disrupt the hsp-hTERT association. Cells were harvested or continuously cultured with 10 *μ*M SC-10 for an additional 24 h. Cellular proteins were extracted for determination of telomerase activity.

Since we have previous demonstrated that telomerase holoprotein integrity is crucial for full enzyme activity (Chang *et al*, 2002), we investigated whether PKC phosphorylation had an effect on holoenzyme integrity. To address this question, cells were exposed to a hsp90 inhibitor, novobiocin (Haendeler *et al*, 2003), to disassociate hsp90 from other proteins. A PKC activator, SC-10 was also used to examine the potential influence of PKC phosphorylation. As shown in Figure 4A, more phosphosubstrate was detected in SC-10-treated proteins, indicating that PKC activity increased after SC-10 treatment. Immunoprecipitation and immunoblot analysis were used to examine the status of protein association. OEC-M1 cells were treated with novobiocin for 24 h. Cells were harvested or continuously cultured with SC-10 for an additional 24 h. As shown in Figure 4B, a hTERT-immunoblotted band was detected after immunoprecipitation by hsp90, indicating the association of these two proteins (lane 1). After specific inhibition of PKC by RNAi, this association was disrupted (lane 2), suggesting that PKC phosphorylation plays a crucial role in maintaining the integrity of the telomerase holoenzyme. The association of hsp90 and hTERT was disrupted (lane 3), however, was restored after induction of PKC function by SC-10 (lane 4), suggesting PKC phosphorylation promotes interaction of hsp90 with hTERT.

To examine whether the maintenance of telomerase holoprotein integrity by PKC is crucial for enzyme function, the enzyme activity was determined after disruption of the hTERT-hsp90 association by novobiocin and its reassociation by PKC activator SC-10. After OEC-M1 cells were treated with novobiocin for 24 h, cells were harvested or continuously cultured with SC-10 for additional 24 h. Telomerase activity was determined. Results were shown in the Figure 4C. Telomerase activity was suppressed after disruption of the holoprotein structure by novobiocin (lane 3), similarly as found in the treatment of PKC-RNAi (lane 2), indicating the telomerase holoprotein integrity was crucial for enzyme activity. However, the novobiocin-inhibited activity was restored by SC-10 (lane 4), consistent with the findings in the previous experiments on protein interaction (Figure 4B). Thus, the role of PKC phosphorylation in telomerase activity appears to occur through promoting telomerase holoenzyme assembly and/or maintaining the holoprotein integrity.

### PKC isoenzymes *α*, *β, δ*, *ε* and *ζ* were overexpressed in tumour samples, correlating with a high level of telomerase activity

**Figure 5:**
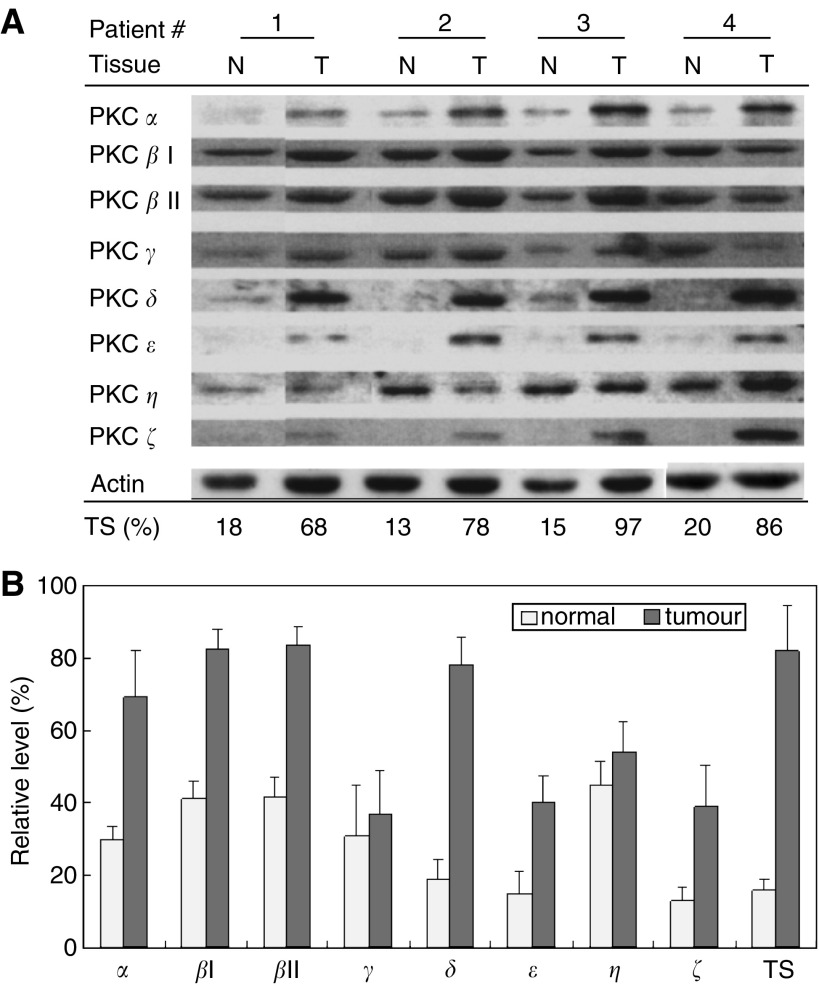
Relative levels of telomerase activity and the expressions of PKC isoenzymes in normal and tumour tissues. Four pairs of normal (N) and tumour (T) tissues from head and neck cancer patients were examined. Each sample is indicated at the top of the figure. (**A**) The protein expression was determined by immunoblot analysis and is indicated at the left of the figure. Actin protein expression was used as an internal control. Telomerase activity in each sample was determined by TRAP-EIA and was normalised with that in the OEC-M1 cell lines. Relative levels of telomerase activity (%TS) are indicated at the bottom of the figure. (**B**) Average of telomerase activity and PKC isoenzyme expression in tumour and normal tissues. After quantitation of the immunoblot densities in each sample, the levels of PKC isoenzymes were normalised with their respective actin level to calculate the relative expression. Average of telomerase activity in each sample was also determined as indicated. ^*^Statistical significance using student *t*-test (*P*<0.05 of two-sided test).

To understand the function of PKC isoenzyme in the carcinogenesis of head and neck cancers and the potential association with telomerase activity, four tumour samples from patients with head and neck squamous cell carcinoma and their respective grossly normal mucosa tissues were obtained for study. PKC isoenzymes were determined by immunoblot analysis and telomerase activity was measured by TRAP-EIA method. Results of PKC isoenzyme expression and the relative level of telomerase activity normalised with that in OEC-M1 cells are shown in Figure 5A. The average quantitative results of each PKC isoenzyme normalised with actin levels and average level of telomerase activity were shown in Figure 5B. Differential concentrations of the various isotypes were found. On average, PKC *α*, *β*I, *β*II, *δ*, *ε* and *ζ* had greater than two-fold overexpression in the tumour samples compared to the normal tissue counterparts, which were correlated with an increase in telomerase activity. This was not true for PKC *γ* or *η*. These clinical data further support our findings that PKC *α*, *β*, *δ*, *ε* and *ζ* participate in the carcinogenesis of head and neck cancer, probably by the way of telomerase activation.

### Inhibition of telomerase through dephosphorylating PKC increases chemosensitivity to cisplatin

**Figure 6:**
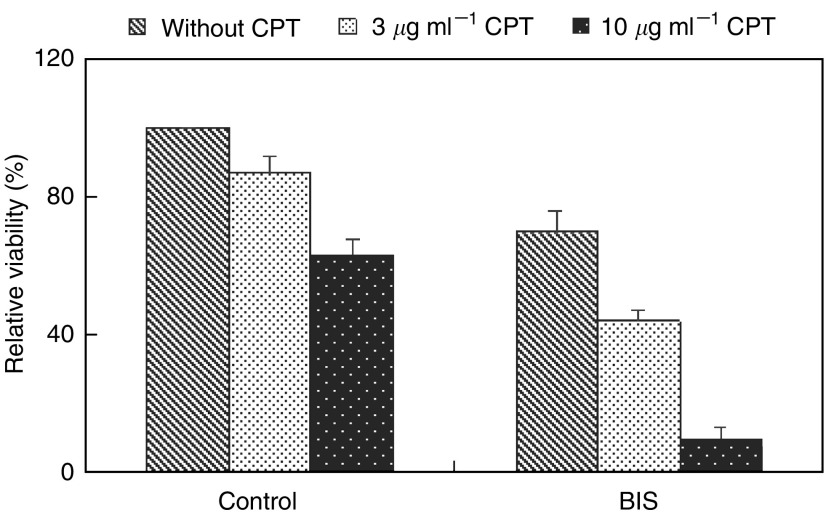
Increased chemosensitivity to cisplatin-induced cell death after inhibition of telomerase activity BIS-induced dephosphorylation of PKC. OEC-M1 cells were treated with 40 *μ*M BIS for 48 h, followed by administration of various amounts of cisplatin (0, 3 or 10 *μ*g ml^−1^) for an additional 12 h. Cell viability was determined using trypan blue staining.

To examine whether the inhibition of telomerase through dephosphorylating PKC influences the chemosensitivity of head and neck cancer cells, OEC-M1 cells were treated with 40 *μ*M BIS for 48 h, followed by administration of various doses of cisplatin (0, 3 or 10 *μ*g ml^−1^) for an additional 12 h. Cell viability was determined to assess the chemosensitivity to cisplatin (Figure 6). There was a minimal effect on cell death at a dose 3 *μ*g ml^−1^ of cisplatin in the control group. There was, however, a significant decrease in cell viability after BIS treatment, an effect more apparent with 10 *μ*g ml^−1^ of cisplatin. At this concentration, 63% cell viability was observed in the control group compared with only 10% of cells surviving with BIS treatment. These results indicate that inhibition of telomerase activity by BIS significantly increases the sensitivity to cisplatin.

## DISCUSSION

In this study, we investigated how PKC regulates telomerase activity in head and neck cancer cells. Possible mechanisms of PKC in the regulation of telomerase activity include enhanced expression of telomerase genes through a PKC-dependent signal pathway, phosphorylation of telomerase subunit proteins, or both. We found that PKC regulation of telomerase activity in these cells occurs through phosphorylation (Figure 2C) but not by influencing the expression of telomerase core subunits (Figure 1). Our RNAi transfection studies revealed that specific PKC isoforms, namely *α*, *β*, *δ*, *ε* and *ζ*, involved in regulating telomerase activity (Figure 2B), which was in agreement with the findings in clinical cancer tissue specimens (Figure 5). *In vitro* phosphorylation experiments further demonstrated that the target of PKC isoenzymes is the hTERT molecule (Figure 3).

Although hTERT is a crucial component of telomerase and subject to regulation, the association of other telomerase subunits, such as chaperone protein hsp90, is required for enzyme activity (Holt *et al*, 1999; Chang *et al*, 2002). The binding of hsp90 to hTERT was been shown to be essential for assembly of the telomerase complex and its activity (Holt *et al*, 1999). However, the underlying mechanism is not fully elucidated. The present study has demonstrated that specific inhibition of PKC by RNAi transfection disrupts the hTERT-hsp90 association (Figure 4), suggesting that PKC affects protein interaction. Furthermore, an hsp90 inhibitor, novobiocin, interfered with hTERT-hsp90 interaction, suggesting hTERT association is in close to the vicinity of novobiocin binding site within hsp90. The protein association was restored after treatment with the PKC activator SC-10, further evidence that PKC phosphorylation results in hTERT-hsp90 interaction, a key function in maintaining the integrity of the telomerase holoenzyme. However, since hsp90 chaperone is known to facilitate the folding and assembling of several proteins, this molecule maybe also involve in interaction with other telomerase subunits, presumably at varying sites other than hTERT binding domain. To summarise our findings, the PKC isoenzymes *α*, *β*I, *β*II, *δ*, *ε* and *ζ* regulate telomerase activity in head and neck cancer cells through phosphorylation of hTERT, a holoenzyme assembly step that is essential telomerase activation and oncogenesis.

While phosphorylation of hTERT is essential for telomerase activation, PKC is likely not the only enzyme responsible for phosphorylating this molecule. Telomerase activity in human breast cancer cells is markedly inhibited by treatment with protein phosphatase 2A (Li *et al*, 1998). Protein kinase Akt also activates telomerase by phosphorylating hTERT in melanoma and epithelial cells (Kang *et al*, 1999; Breitschopf *et al*, 2001). A potential mechanism of telomerase regulation through hTERT phosphorylation linked to nuclear localisation of the enzyme has been reported in T lymphocytes during cell activation (Liu *et al*, 2001). Recently, the association of hTERT with hsp90 and Akt in concert with hTERT phosphorylation has been found in human endothelial cells, suggesting that Akt phosphorylation also plays a role in assembly of the holoprotein (Haendeler *et al*, 2003). Our results, demonstrate the mechanism by which PKC functions in maintaining telomerase holoenzyme integrity and hence activation in head and neck cancer cells. Given the common requirement of telomerase activity in cancer cells because of the enzymes pleiotropic functions, it is not surprising that hTERT appears to be regulated by many kinases, PKC and Akt among them. It may well be that the principle enzymes involve in activation of telomerase vary with specific tissues or specific cellular states and perhaps participate in various molecular mechanisms.

Our finding that PKC isoenzymes *α*, *β*, *δ*, *ε* and *ζ* are correlated with increased telomerase activity in clinical head neck tumour samples (Figure 5). Although the data set is few, these results implicate the significance of these molecules in carcinogenesis in head and neck cancer. PKC isoenzymes have been found to display variable expression profiles depending on specific cancer type. For example, PKC*α* and *β* has shown over-expressed in both high grade of prostate, gastrointestinal tract and head neck cancers (Koren *et al*, 2004; Lahn *et al*, 2004; Martinez-Gimeno *et al*, 1995). In contrast, hepatocellular and breast cancer display a downregulation of PKC*α*, and bladder cancer shows a down regulation of PKC*β* (Tsai *et al*, 2000; Kerfoot *et al*, 2004; Varga *et al*, 2004). However, vast majority of the studies have demonstrated the functions of PKC *α* and *β* associated with the increased invasion and proliferation activity, while the inhibition of these PKC isoenzymes effectively reverses the malignant phenotype (Hanauske *et al*, 2004; Jiang *et al*, 2004; Koivunen *et al*, 2004). Our finding that PKC isoenzymes *α* and *β* overexpressed in head neck cancers is in agreement with most findings that these two PKC isoenzymes play positive regulatory roles in carcinogenesis. Regarding PKC*δ*, the expression of this molecule in cancers has not been extensively studied. However, similarly to classical PKC (*α*, *β*), PKC*δ* was shown either upregulated (hepatocellular cancer) or downregulated (urinary bladder cancer) in various cancer tissues (Tsai *et al*, 2000; Varga *et al*, 2004). Our finding that PKC*δ* upregulated in cancer tissues in head neck cancer was consistent with the finding in hepatoma. The most important function of PKC*δ* is thought to promote apoptosis in response to DNA damage or oxidative stress in cells (Basu, 2003). This enzyme activity is known to result in many proapoptotic signals such as increased expression and stability of p53, release of mitochondrial cytochrome *c*, or activation of c-Abl (Majumder *et al*, 2000; Sun *et al*, 2000; Abbas *et al*, 2004). Since our head neck cancer patients have betel quid chewing habit (Chen *et al*, 2003), a potent carcinogen of oral cancer, our finding of PKC*δ* overexpressed in the cancer patients may be explained triggered by the chemical stimulus. Aside from apoptotic induction, in the present study, PKC*δ* was also found activation of hTERT and correlation with telomerase activity in tissues. Therefore, PKC*δ* may function as a molecular sensor, which participates in the transformation of cell to be immortalised under survival conditions and executes the death of severe damaged cells. As for PKC *ε* and *ζ*, although their cellular function still obscure, their expressions have been reported in various cancers. PKC*ε* has been shown upregulated in head neck, brain and prostate cancers (Xiao *et al*, 1994; Martinez-Gimeno *et al*, 1995; Sharif and Sharif, 1999; Koren *et al*, 2004), whereas downregulated in pancreatic cancer (Evans *et al*, 2003). PKC*ζ* have been shown upregulated in brain, liver and bladder cancers (Xiao *et al*, 1994; Tsai *et al*, 2000; Varga *et al*, 2004). Despite various reports, most of the studies favour the roles of PKC *ε* and *ζ* in the positive regulation of cancer growth, which are consistent with our findings of overexpression in head neck cancer tissues. Perhaps, the reactivation of telomerase through PKC phosphorylation by these isoenzymes may be one of the mechanisms leading to the carcinogenesis.

Understanding the mechanisms underlying oncogenesis has wide-ranging implications for targeting the treatment of cancer. In particular, treatment strategies directed at tumour-specific functions, such as activation of telomerase, should minimise cytotoxic effects on normal cells. PKC dephosphorylation enhanced the chemosensitivity to cisplatin of cancer cells *in vitro* (Figure 6) is an example of such an approach. Our study provides a foundation for further investigation into the manipulation of telomerase activity as a potential therapeutic modality.

## Figures and Tables

**Table 1:** List of RNAi oligonucleotides for each PKC isoenzyme

**Gene**	**Sequence**
PKC *α*	5′-CGACTGGGAAAAACTGGAGAAGCTTGTCCAGTTTTTCCCAGTCG-3′
	5′-GGATCGACTGGGAAAAACTGGACAAGCTTCTCCAGTTTTTCCCAGTCG-3′
	
PKC *β*	5′-GAAGATGAACTCTTCCAAGAAGCTTGTTGGAAGAGTTCATCTTC-3′
	5′-GGATGAAGATGAACTCTTCCAACAAGCTTCTTGGAAGAGTTCATCTTC-3′
	
PKC *γ*	5′-TCTTTCCCCAGAGGCTCCGAAGCTTGGGAGCCTCTGGGGAAAGA-3′
	5′-GGATTCTTTCCCCAGAGGCTCCCAAGCTTCGGAGCCTCTGGGGAAAGA-3′
	
PKC *δ*	5′-GTGGTCCTGATCGACGACGAAGCTTGGTCGTCGATCAGGACCAC-3′
	5′-GGATGTGGTCCTGATCGACGACCAAGCTTCGTCGTCGATCAGGACCAC-3′
	
PKC *ε*	5′-GATGACGTGGACTGCACAGAAGCTTGTGTGCAGTCCACGTCATC-3′
	5′-GGATGATGACGTGGACTGCACACAAGCTTCTGTGCAGTCCACGTCATC-3′
	
PKC *η*	5′-GGAACTTTCAGATATCAAGAAGCTTGTTGATATCTGAAAGTTCC-3′
	5′-GGATGGAACTTTCAGATATCAACAAGCTTCTTGATATCTGAAAGTTCC-3′
	
PKC *ζ*	5′-TACACTCCTGCTTCCAGAGAAGCTTGTCTGGAAGCAGGAGTGTA-3′
	5′-GGATTACACTCCTGCTTCCAGACAAGCTTCTCTGGAAGCAGGAGTGTA-3′
